# Prevalence of *Leishmania* infection in three communities of Oti Region, Ghana

**DOI:** 10.1371/journal.pntd.0009413

**Published:** 2021-05-27

**Authors:** Richard Akuffo, Michael Wilson, Bismark Sarfo, Naiki Attram, Mba-Tihssommah Mosore, Clara Yeboah, Israel Cruz, Jose-Antonio Ruiz-Postigo, Daniel Boakye, Javier Moreno, Francis Anto

**Affiliations:** 1 Noguchi Memorial Institute for Medical Research, University of Ghana, Accra, Ghana; 2 School of Public Health, University of Ghana, Accra, Ghana; 3 U.S. Naval Medical Research Unit No. 3, Ghana Detachment, Accra, Ghana; 4 National School of Public health, Instituto de Salud Carlos III, Madrid, Spain; 5 Department of Control of Neglected Tropical Diseases, World Health Organization, Geneva, Switzerland; 6 WHO Collaborating Center for Leishmaniasis, Instituto de Salud Carlos III, Madrid, Spain; Federation University Australia, AUSTRALIA

## Abstract

**Background:**

Leishmaniasis is a neglected tropical disease caused by parasites of the genus *Leishmania* and is transmitted by various species of female phlebotomine sand flies. The first report of cutaneous leishmaniasis (CL) in Ghana refer to a cluster of cases in 1999–2003 in the Ho municipality of the Volta Region. We conducted an epidemiological assessment in the Oti Region, encouraged by recent reports of potential cases of CL.

**Methodology/Principal findings:**

Using a cross-sectional study design, the exposure to *Leishmania* was investigated in three communities of the Oti Region based on the leishmanin skin test (LST). LST results for 3,071 participants comprising 1091, 848, and 1132 persons from the communities of Ashiabre, Keri, and Sibi Hilltop, indicated an overall prevalence of exposure to *Leishmania* infection of 41.8% and individual community prevalence of 39.4%, 55.1%, and 34.2% respectively. Being male [AOR = 1.27; CI: 1.09, 1.49], and living in Keri [AOR = 1.83; CI: 1.43, 2.34] were associated with an increase in the odds of exposure to *Leishmania*. Being 5–10 years old [AOR = 1.48; CI: 1.06, 2.05], 11–17 years old [AOR = 2.03; CI: 1.45, 2.85], 18–40 years old [AORR = 2.83; CI: 1.81, 4.43] and 41–65 years old [AOR = 5.08; CI: 2.98, 8.68] were also significantly associated with increased odds of being exposed to *Leishmania*.

**Conclusions/Significance:**

This study demonstrated exposure to *Leishmania* in the study communities and also identified associated factors. Future efforts aimed at reducing exposure to *Leishmania* infection in the study area should take the associated factors into consideration.

## Author summary

Leishmaniasis is a neglected vector borne disease caused by parasites of the genus *Leishmania* and is endemic in over 98 countries globally. Two broad categories of leishmaniasis exist: visceral leishmaniasis (VL) and cutaneous leishmaniasis (CL). The first report of CL in Ghana was from a cluster of cases in 1999–2003 in the Ho municipality of the Volta Region [which has since the year 2019 been divided into Volta and Oti Regions]. However, information on the prevalence of *Leishmania* infection in the affected communities of Ghana is limited. Using a cross-sectional study design, this study described prevalence of exposure to *Leishmania* in three communities of the Oti Region based on the leishmanin skin test (LST), following reports of potential cases of CL in that area. In addition, factors associated with an increase in the odds of exposure to *Leishmania* were also investigated. Demonstration of exposure to, as well as factors associated with *Leishmania* infection in communities of the Oti Region of Ghana suggests a need for further investigation of other related factors such as prevalence of CL and possible vectors and reservoirs of the disease.

## Introduction

Leishmaniasis is a neglected vector borne disease caused by parasites of the genus *Leishmania* and is endemic in over 98 countries with 350 million people estimated to be at risk of contracting the disease globally [1,2]. Natural transmission of the *Leishmania* parasites to humans and other mammals occurs through the bite of various species of infected female phlebotomine sand flies belonging to the genus *Phlebotomus* in the Old World and the following genera in the New World: *Lutzomyia*, *Psychodopygus* and *Nyssomyia*. Majority of the *Leishmania* infections in humans are transmissible only from animals [zoonotic leishmaniasis] but some can be spread between humans (anthroponotic leishmaniasis) [3–7].

Depending on the area of localization of the parasite in mammalian tissues, two broad categories of leishmaniasis exist: visceral and cutaneous, with cutaneous leishmaniasis (CL) being the most common. Globally, it is estimated that between 0.7 to 1.3 million new cases of CL are reported every year [1–3,8].

Although lesions due to CL are often self-healing, this could take up to six months. Secondary bacterial infections, may increase tissue destruction which can lead to permanent scarring [3,7].

A localized outbreak of skin ulcers suspected to be cases of CL was first reported in Ghana from the Ho municipality of the Volta Region in 1999 based on the identification of *Leishmania* amastigotes in some skin lesion biopsies obtained from the patients and examined using microscopy [9]. In 2002, molecular typing revealed *Leishmania major* causing CL in a patient from the Ho municipality, Volta Region [10]. A follow up study involving nine biopsies from five suspected CL cases in 2006 and one biopsy from another suspected CL case in 2007 from Taviefe, a community located about 10 km north of Ho, however, did not confirm *L*. *major*. Instead, uncharacterized species of *Leishmania* were observed [11].

An additional study in some other parts of the Ho municipality successfully cultured and obtained three isolates from individuals suspected with active cutaneous leishmaniasis. Using DNA sequencing and phylogenetic analysis, the isolates were confirmed to be members of the *Leishmania enriettii* complex, an emerging new subgenus of *Leishmania* parasites [12]. Also, *L*. *tropica*, and *L*. *major* DNA have been identified in *Sergentomyia* sand flies sampled from the Ho municipality [13]. These results suggest a complex epidemiology of CL in the region.

Despite this, information on the prevalence of *Leishmania* infection in the affected communities of Ghana is limited possibly due to the absence of a surveillance system for leishmaniasis in Ghana [9]. This study was therefore conducted in three communities of the Oti Region [which until December, 2018 was part of the Volta Region] to investigate the prevalence of *Leishmania* infection following reports of cases of skin ulcers suspected to be CL, using the leishmanin skin test (LST).

The leishmanin skin test (LST), also known as the Montenegro skin test (MST) is an intradermal test that measures delayed-type hypersensitivity response to *Leishmania* antigen, and is a useful method for detecting cell-mediated immunity against *Leishmania* [14,15].

The LST becomes positive after an asymptomatic *Leishmania* infection, during active CL, and after healing of CL ulcers. A positive LST is defined as skin reaction [induration] ≥5 mm with sensitivity of the LST estimated at between 86.4% to 100%. The LST is therefore a useful tool in epidemiological surveys but has limited value in the diagnosis of CL, as it cannot distinguish between past or active infections. It is more intended as a marker of exposure to *Leishmania* [14–17]

## Materials and methods

### Ethics statement

Ethical approval to conduct this study was obtained from the ethics review committee of the Ghana Health Service (GHS-ERC006/08/18). Written informed consent was obtained from all study participants. For participants under 18 years, written consent was obtained from a parent or guardian.

### Study design

Using a cross-sectional study design, this study was conducted in three communities of the Oti region of Ghana from October to December, 2018. Prevalence of *Leishmania* infection as well as factors (individual level, household level, and community of residence) associated with *Leishmania* infection among study participants were investigated based on the leishmanin skin test and a structured interviewer administered questionnaire [18].

### Study area

This study was conducted in the following three communities: Ashiabre, Keri, and Sibi Hilltop. Ashiabre is in the Tutukpene sub-district of the Nkwanta South municipality while Keri is in the Keri sub-district of the municipality. Sibi Hilltop is in the Sibi sub-district of the Nkwanta North district of the region.

The population of Nkwanta South municipality is estimated to be 117,878 with males constituting 49.6% of the population. Covering a land area of approximately 2733 km^2^, the Nkwanta South municipality is located between latitudes 7^o^ 30’ and 8^o^ 45’ North and longitude 0^o^ 10’ and 0^o^ 45’East [19].

The population of the Nkwanta North district is estimated to be 64,553 with males constituting 50.2% of the population. The district is located between Latitude 7°30’N and 8°45’N and Longitude 0°10’W and 045’E. It shares boundaries with Nkwanta South municipality to the south, Nanumba South to the north, Republic of Togo to the east, and Kpandai District to the west [20].

### Inclusion criteria

Eligible study participants were residents in the study community for ≥ 12 months, aged between 2 to 65 years (inclusive).

### Sample size consideration

Assuming a 0.05 seropositivity of delayed-type hypersensitivity to *Leishmania* antigen in the study area, acceptable difference of 0.0175, an alpha error of 0.05, and a design effect of 1.5, a minimum sample of 834 persons was required for LST screening [21].

### Sampling method

Of 15 communities in the Oti Region visited by the study team prior to study initiation, at least 3 potential cases of CL were observed in 8 communities.

The three study communities were selected from among the eight using a simple random selection without replacement approach.

Using a sorted list of households in each study community, 200 households [with an average of 5–7 persons per household] were selected for study inclusion using a systematic sampling approach. For this study, a household was defined as a person or a group of persons, who live together in the same house or compound and share the same house-keeping arrangements. The head of each household was defined as a male or female member of the household recognised as such by the other household members. The head of a particular household is generally the person with economic and social responsibility for the household. As a result, household relationships were defined with reference the household head [22]. The community household list was obtained for each study community based on a household census.

The total number of households in Ashiabre, keri, and Sibi Hilltop were 945, 795, and 1184 respectively.

The systematic sampling approach used is described below.

Using the sorted list of households in each study community, a sampling interval I was determined, where I = N/n with N being the number of households in the study community [number of units forming the sample frame] and n was the number of households to be selected [200 for each study community]. The I was rounded to 2 decimal places.

Using Microsoft excel, the RANDBETWEEN command was used to generate a random decimal integer R between 0 and 1 rounded up to two decimal points. The sequence of households that were selected in each study community were R*I, R*I + I, R*I +2*I, R*I +3*I,….R*I + [n-1]*I, each rounded up to the next highest whole number [23].

With the assistance of community-based volunteers, the selected households were identified after which all members of the selected households aged 2 to 65 years were invited to participate in the study, using a door-to-door invitation approach. Because the invitation to participate in the study was extended to households, a household was not included in the study if the household head declined to allow his or her household to participate in the study. However, the agreement of the household head did not make it compulsory for every household member of age 2 to 65 years to participate in the study. Each household member was given the opportunity to go through the informed consent process to decide whether they wish to participate or not.

### Pre-study training

Prior to the commencement of field data collection, study team members were taken through a one-week training session comprising in-class training, break out discussion sessions, and field testing of the study questionnaires in a community in the Nkwanta South municipality [the main Nkwanta township].

The training sessions covered all aspects of the study procedures such as informed consent process, questionnaire administration, and administration of the leishmanin skin test. The trainees were also taken through a simulation of all study procedures.

The field team was comprised of health workers (community health nurses often involved in administration of vaccinations such as bacille Calmette-Guérin (BCG), hospital-based nurses and public health officers) and community-based volunteers.

### Administration of leishmanin skin test

**Fig 1:**
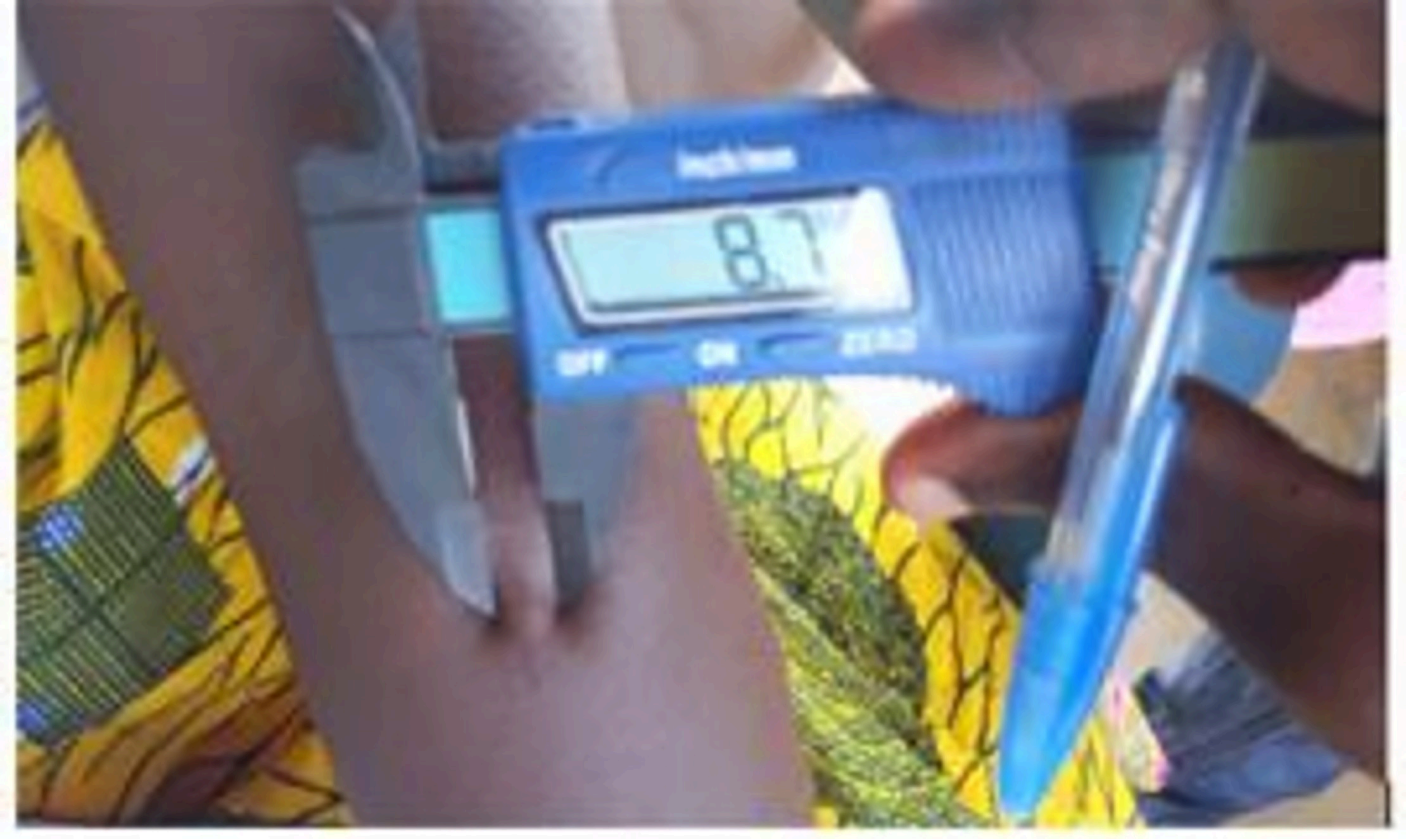
Induration on skin 48–72 hours after LST placement and measurement.

All study participants were invited to participate in the LST survey to establish prevalence of *Leishmania* infection. The leishmanin reagent used for this study was obtained from the Pasteur Institute of Iran and was composed mainly of the following: promastigotes of *Leishmania major* (MRHO/IR/75/ER strain), phosphate buffered saline, and thimerosal. The leishmanin used was lot number 128, manufactured in February 2016, and expected to expire in February 2021. The sterile aqueous suspension of leishmanin used for this study was stored in a refrigerator at 2–8 degrees Celsius with cold chain maintained during field work using ice packs. Details of the LST procedure are provided below:

A 0.1 mL of LST suspension was injected intradermally into the volar aspect of each study participant’s left forearm using new sterile tuberculin syringes. Between 48 and 72 hours post-LST placement, the delayed-type hypersensitivity (DTH) reaction was assessed by averaging the greatest diameter of induration and the diameter of induration perpendicular to it, measured in millimeters using calipers. A positive LST was defined as induration ≥5 mm (Fig 1) [14].

### Data management and analysis

Data were managed using Microsoft Access software version 2013 and analyzed using STATA software version 14. All statistical tests were performed at a 95% confidence level.

Prevalence of LST positivity was described. Logistic regression model [simple and multiple] was used to evaluate association between various factors and *Leishmania* infection as observed using the LST, in the study communities. Factors with p value less than 0.05 in the bivariate analysis were included in the multiple logistic regression analysis.

The factors compared with LST positivity included age of study participant, sex of participant, number of scars, number of skin ulcers, family history of CL, use of bed nets, being at open field at dawn or sunset, sleeping near forest or farm fields, and sleeping in rooms or places with open windows without screen. Additional factors included were frequency of mosquito bites, having bites from other insects, having contact with dogs, having contact with goats, having contact with other domestic animals, spraying bedroom with insecticide in the last six months, and use of long sleeves, use of mosquito repellents. These variables were obtained from the administration of a structured questionnaire.

Odds ratios for all variables included in the multiple logistic regression analysis were adjusted for all covariates included in the model as well as for clustering at the household level using the vce (cluster clustvar) command in Stata statistical software version 14.

## Results

Of 600 households (200 in each study community) invited to participate in this study, a total of 587 households comprising 189 (32.2%), 200 (34.1%), and 198 (33.7%) from Ashiabre, Keri and Sibi Hilltop respectively, agreed to be part of this study and were included. The study households had a total of 3718 members out of which 3,440 (92.5%) consisting of 1,194, 941, 1305 from Ashiabre, Keri, and Sibi Hilltop respectively were enrolled in the study.

The average household size was 6.3 with a range of 1 to 18 household members. Ashiabre and Sibi Hilltop had an average household size of 7 while Keri had an average household size of 5.

A total of 3071 persons comprising 1091, 848, and 1132 participants from Ashiabre, Keri, and Sibi Hilltop respectively were screened using the LST procedure. Of these, 1483 (48.3%) were males and 345 (11.2%) were under five years old (Table 1).

**Table 1:** Summary of study participants by age, sex, and community of residence.

Characteristic	Study Community			
	Ashiabre	Keri	Sibi Hilltop	Total
Mean age in years [SD]	18.6 (±15.2)	19.5 (±15.5)	17.9 (±14.2)	18.6 (±14.9)
Age group, n [%]				
<5 years	137 (12.6)	86 (10.1)	122 (10.8)	345 (11.2)
5–10 years	289 (26.5)	249 (29.4)	330 (29.2)	868 (28.3)
11–17 years	277 (25.4)	189 (22.3)	297 (26.2)	763 (24.9)
18–40 years	251 (23.0)	210 (24.8)	268 (23.7)	729 (23.7)
41–65 years	137 (12.6)	114 (13.4)	115 (10.2)	366 (11.9)
Sex, n [%]				
Male	523 (47.9)	381 (44.9)	579 (51.2)	1483 (48.3)
Female	568 (52.1)	467 (55.1)	553 (48.9)	1588 (51.7)
**Total**	**1091 (100)**	**848 (100)**	**1132 (100)**	**3071 (100)**

Prevalence of *Leishmania* infection observed at Ashiabre and Keri was 39.4% and 55.1% respectively [Table 2]. Prevalence among males at Ashiabre was 43.6% and 35.6% among females in the same community. Among both males and females at Ashiabre, prevalence of *Leishmania* infection increased with age from 18.8% and 19.1% among males and females under five respectively to 70.8% and 55.6% among males and females 41–65 years old, respectively. Cumulatively, prevalence of *Leishmania* infection at Ashiabre increased from 19.0% among children under five, to 62.8% among adults 41–65 years old (Table 2).

**Table 2:** LST prevalence by age and sex at Ashiabre and Keri.

Community	Sex	Age	Number of household	LST Prevalence		P value
			members screened	n (%)	95% CI	
Ashiabre	Males	< 5 years	69	13 (18.8)	(11.1, 30.1)	<0.001
		5–10 years	157	56 (35.7)	(28.5, 43.5)	
		11–17 years	147	62 (42.2)	(34.4, 50.4)	
		18–40 years	85	51 (60.0)	(49.1, 70.0)	
		41–65 years	65	46 (70.8)	(58.3, 80.7)	
		Subtotal	523	228 (43.6)	(39.4, 47.9)	
	Females	< 5 years	68	13 (19.1)	(11.3, 30.5)	<0.001
		5–10 years	132	28 (21.2)	(15.0, 29.1)	
		11–17 years	130	42 (32.3)	(24.7, 40.9)	
		18–40 years	166	79 (47.6)	(40.0, 55.3)	
		41–65 years	72	40 (55.6)	(43.7, 66.8)	
		Subtotal	568	202 (35.6)	(31.7, 39.6)	
	Total	< 5 years	137	26 (19.0)	(13.2, 26.5)	<0.001
		5–10 years	289	84 (29.1)	(24.1, 34.6)	
		11–17 years	277	104 (37.5)	(32.0, 43.4)	
		18–40 years	251	130 (51.8)	(45.6, 58.0)	
		41–65 years	137	86 (62.8)	(54.3, 70.5)	
		Total	1091	430 (39.4)	(36.5, 42.4)	
Keri	Males	< 5 years	41	10 (24.4)	(13.3, 40.4)	<0.001
		5–10 years	134	62 (46.3)	(37.9, 54.8)	
		11–17 years	97	55 (56.7)	(46.5, 66.3)	
		18–40 years	61	43 (70.5)	(57.6, 80.8)	
		41–65 years	48	41 (85.4)	(71.8, 93.1)	
		Subtotal	381	211 (55.4)	(50.3, 60.3)	
	Females	< 5 years	45	10 (22.2)	(12.1, 37.2)	<0.001
		5–10 years	115	38 (33.0)	(25.0, 42.3)	
		11–17 years	92	57 (62.0)	(51.5, 71.4)	
		18–40 years	149	95 (63.8)	(55.7, 71.2)	
		41–65 years	66	56 (84.9)	(73.7, 91.8)	
		Subtotal	467	256 (54.8)	(50.3, 59.3)	
	Total	< 5 years	86	20 (23.3)	(15.4, 33.5)	<0.001
		5–10 years	249	100 (40.2)	(34.2, 46.4)	
		11–17 years	189	112 (59.3)	(52.0, 66.1)	
		18–40 years	210	138 (65.7)	(59.0, 71.9)	
		41–65 years	114	97 (85.1)	(70.2, 90.6)	
		Total	848	467 (55.1)	(51.7, 58.4)	

At Keri, prevalence of *Leishmania* infection among males was 55.4% while a prevalence of 54.8% was observed among females. Among both males and females at Keri, prevalence of infection increased with age from 24.4% and 22.2% among males and females under five respectively to 85.4% and 84.9% among adult males and females in the 41–65 years old group, respectively. Cumulatively, prevalence of infection at Keri increased from 23.3% among children under five to 85.1% among adults in the 41–65 years group (Table 2).

Table 3 summarizes prevalence of *Leishmania* infection at Sibi Hilltop (34.2%) as well as the cumulative prevalence of infection for all study sites (41.8%). At Sibi Hilltop, prevalence of infection among males was 35.1% while it was 33.3% among females. This increased from 28.8% and 30.2% among males and females under five respectively to 50.8% and 53.7% among adult males and females in the 41–65 years, respectively. Cumulatively, prevalence of infection at Sibi Hilltop increased from 29.5% among children under five to 52.2% among adults in the 41–65 years group.

**Table 3:** LST prevalence by age and sex at Sibi Hilltop and cumulatively for all study sites.

Community	Sex	Age	Number of household	LST Prevalence		P value
			members screened	n (%)	95% CI	
Sibi Hilltop	Males	< 5 years	59	17 (28.8)	(18.5, 42.0)	0.007
		5–10 years	196	67 (34.2)	(27.8, 41.2)	
		11–17 years	165	46 (27.9)	(21.5, 35.3)	
		18–40 years	98	42 (42.9)	(33.3, 53.0)	
		41–65 years	61	31 (50.8)	(38.1, 63.4)	
		Subtotal	579	203 (35.1)	(31.3, 39.1)	
	Females	< 5 years	63	19 (30.2)	(19.9, 42.9)	<0.001
		5–10 years	134	33 (24.6)	(18.0, 32.7)	
		11–17 years	132	35 (26.5)	(19.6, 34.8)	
		18–40 years	170	68 (40.0)	(32.8, 47.6)	
		41–65 years	54	29 (53.7)	(40.0, 66.8)	
		Subtotal	553	184 (33.3)	(29.5, 37.3)	
	Total	< 5 years	122	36 (29.5)	(22.0, 38.3)	<0.001
		5–10 years	330	100 (30.3)	(25.6, 35.5)	
		11–17 years	297	81 (27.3)	(22.5, 32.7)	
		18–40 years	268	110 (41.0)	(35.3, 47.1)	
		41–65 years	115	60 (52.2)	(42.9, 61.3)	
		Total	1132	387 (34.2)	(31.5, 37.0)	
Total	Males	< 5 years	169	40 (23.7)	(17.8, 30.7)	<0.001
		5–10 years	487	185 (38.0)	(33.8, 42.4)	
		11–17 years	409	163 (39.9)	(35.2, 44.7)	
		18–40 years	244	136 (55.7)	(49.4, 61.9)	
		41–65 years	174	118 (67.8)	(60.4, 74.4)	
		Subtotal	1483	642 (43.3)	(40.8, 45.8)	
	Females	< 5 years	176	42 (23.9)	(18.1, 30.8)	<0.001
		5–10 years	381	99 (26.0)	(21.8, 30.6)	
		11–17 years	354	134 (37.9)	(32.9, 43.0)	
		18–40 years	485	242 (49.9)	(45.4, 54.4)	
		41–65 years	192	125 (65.1)	(58.0, 71.6)	
		Subtotal	1588	642 (40.4)	(38.0, 42.9)	
	Total	< 5 years	345	82 (23.8)	(19.6, 28.6)	<0.001
		5–10 years	868	284 (32.7)	(29.7, 35.9)	
		11–17 years	763	297 (38.9)	(35.5, 42.4)	
		18–40 years	729	378 (51.9)	(48.2, 55.5)	
		41–65 years	366	243 (66.4)	(61.4, 71.1)	
		Total	3071	1284 (41.8)	(40.1, 43.6)	

**Fig 2:**
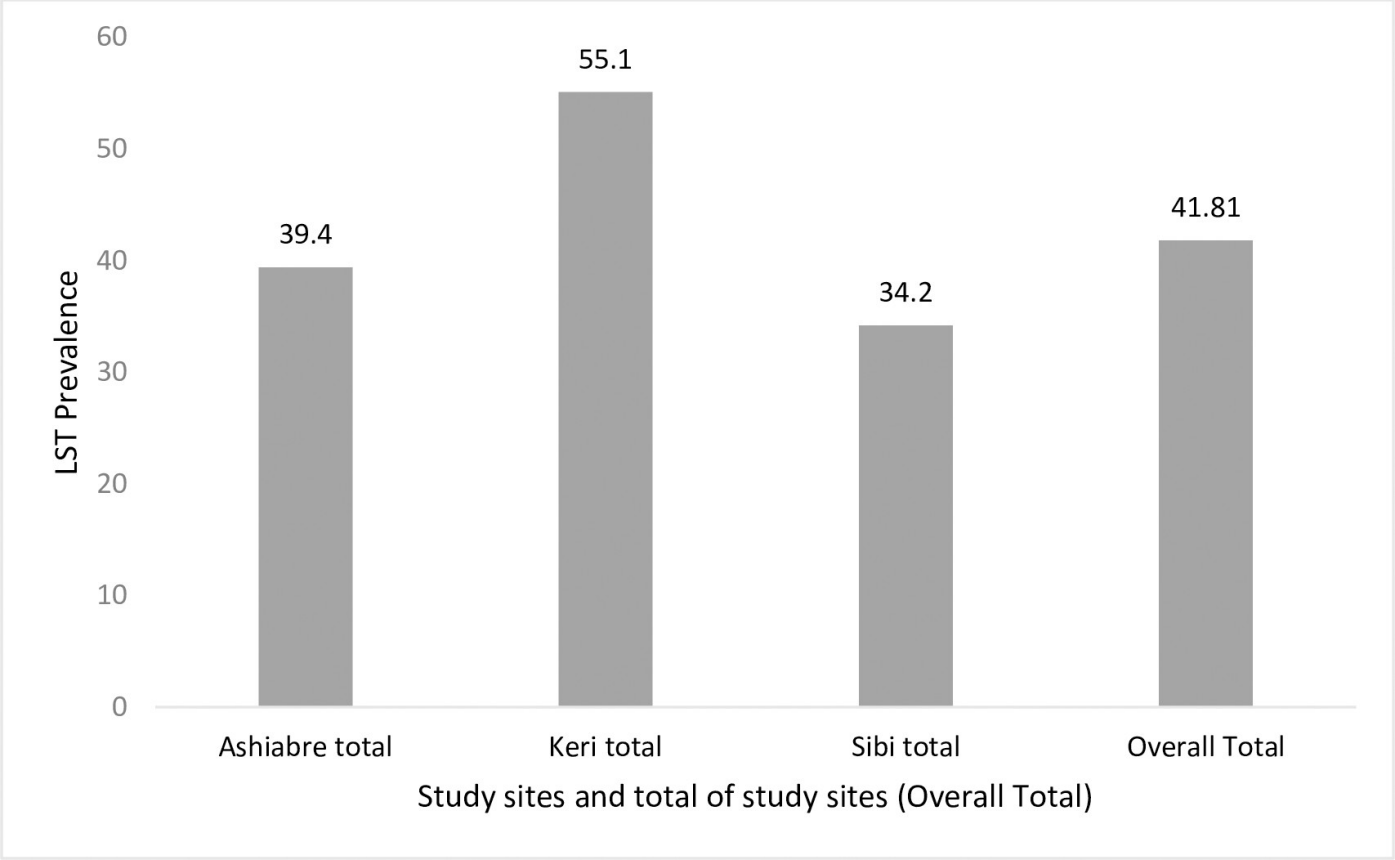
Prevalence of *Leishmania* infection at various study sites and cumulatively.

For all the study communities, a significant association was observed between *Leishmania* infection and age of study participants with the prevalence of infection observed to increase with age. While a cumulative prevalence of 41.8% was observed, prevalence was 23.8%, 32.7%, 38.9%, 51.9%, and 66.4% among those aged <5years, 5–10 years, 11–17 years, 18–40 years, and 41–65 years respectively (Table 3). A summary of the prevalence of *Leishmania* infection across the study communities and cumulatively is presented in Fig 2.

LST positivity was significantly associated with the study community, age of the study participants, and sex of the study participants. Compared to persons in Ashiabre, participants in Keri [AOR = 1.83; 95% CI: 1.43, 2.34] had higher odds of being LST positive. Compared to children under 5 years, participants 5–10 years [AOR = 1.48; 95% CI: 1.06, 2.05], those 11–17 years [AOR = 2.03; 95% CI: 1.45, 2.85], those 18–40 years [AOR = 2.83; 95% CI: 1.81, 4.43] and participants aged 41–65 years [AOR = 5.08; 95% CI: 2.98, 8.68] had higher odds of being LST positive, with the odds increasing with age. Compared to females, males [AOR = 1.27; 95% CI: 1.09, 1.49] had higher odds of being LST positive. Never using insecticide treated bed net (ITN) or use of ITN sometimes was not significantly associated with being LST positive when compared with those who used ITN often (Table 4).

**Table 4:** Factors associated with LST positivity.

Characteristics	Categories	Frequency	Number and proportion LST positive, n(%)	Crude OR (95% CI)	P value	AOR (95% CI)	P value
Study community							
	Ashiabre	1091	430 (39.4)	(Reference)		(Reference)	
	Keri	848	467 (55.1)	1.88 (1.57, 2.26)	<0.001	1.83 (1.43, 2.34)	<0.001*
	Sibi	1132	387 (34.2)	0.80 (0.67, 0.95)	0.011	0.65 (0.50, 0.85)	0.002
	Subtotal	3071	1284 (41.8)				
Person’s age							
	< 5 years	345	82 (23.8)	(Reference)		(Reference)	
	5–10 years	868	284 (32.7)	1.56 (1.17, 2.08)	0.003	1.48 (1.06, 2.05)	0.020*
	11–17 years	763	297 (38.9)	2.04 (1.51, 2.76)	<0.001	2.03 (1.45, 2.85)	<0.001*
	18–40 years	729	378 (51.9)	3.45 (2.57, 4.65)	<0.001	2.83 (1.81, 4.43)	<0.001*
	41–65 years	366	243 (66.4)	6.34 (4.51, 8.91)	<0.001	5.08 (2.98, 8.68)	<0.001*
	Subtotal	345	1284 (41.8)				
Person’s sex							
	Female	1588	642 (40.4)	(Reference)		(Reference)	
	Male	1483	642 (43.3)	1.12 (0.97, 1.29)	0.121	1.27 (1.09, 1.49)	0.002*
	Subtotal	3071	1284 (41.8)				
Number of scars on skin							
	No scars	555	232 (41.8)	(Reference)		(Reference)	
	1–3 scars	615	224 (36.4)	0.77 (0.61, 0.98)	0.035	0.99 (0.76, 1.28)	0.923
	4–6 scars	582	226 (38.8)	0.86 (0.67, 1.09)	0.215	1.07 (0.80, 1.42)	0.665
	7–9 scars	482	195 (40.5)	0.90 (0.70, 1.16)	0.424	1.07 (0.78, 1.48)	0.674
	> = 10 scars	837	407 (48.6)	1.29 (1.03, 1.61)	0.028	1.28 (0.97, 1.69)	0.084
	Subtotal	3071	1284 (41.8)				
Family CL history							
	No family history	825	341 (41.3)	(Reference)		(Reference)	
	1–3 persons with CL history	1874	772 (41.2)	0.99 (0.84, 1.17)	0.946	1.01 (0.83, 1.23)	0.946
	4–6 persons with CL history	307	139 (45.3)	1.17 (0.90, 1.53)	0.233	1.40 (0.94, 2.06)	0.094
	7–9 persons with CL history	25	13 (52.0)	1.54 (0.69, 3.41)	0.29	1.39 (0.66, 2.91)	0.382
	> = 10 persons	40	19 (47.50)	1.28 (0.68, 2.43)	0.441	1.49 (0.84, 2.65)	0.170
	Subtotal	3071	1284 (41.8)				
Use of bed nets							
	Often	2128	885 (41.6)	(Reference)		(Reference)	
	Sometimes	679	306 (45.1)	1.14 (0.96, 1.36)	0.137	0.85 (0.68, 1.07)	0.163
	Never	264	93 (35.2)	0.89 (0.64, 1.24)	0.490	0.80 (0.55, 1.15)	0.227
	Subtotal	3071	1284 (41.8)				

## Discussion

Using the LST, an overall prevalence of 41.8% was determined for *Leishmania* infection, with individual community *Leishmania* prevalence of 39.4%, 55.1%, and 34.2% observed in Ashiabre, Keri, and Sibi Hilltop respectively. This result therefore indicates the presence cutaneous leishmaniasis in the region. However, culture or molecular sequencing confirmation from infected ulcers is required to identify the infecting species.

Data on LST positivity obtained from this study compares with that observed in some other African countries such as Mali where community based studies using LST observed *Leishmania* prevalence as high as 49.9% in certain communities with increase of prevalence associated with age groups such that in a certain community (Diema), *Leishmania* prevalence increased from 13.8% to 88% for age groups of 2–5 years and 41–56 years respectfully [24].

A systematic review of some studies conducted in Mali further observed an overall prevalence of *Leishmania* infection using LST to be 22.1% [25]. Country wide estimates for *Leishmania* infection in Ghana is currently not available. Data obtained from this study on *Leishmania* infection therefore augments existing data on leishmaniasis in Ghana and Africa as a whole where a general paucity of data on leishmaniasis exists [1].

As has been observed in other studies, this study detected significant association between increase in the prevalence of LST positivity and increase in age across the study communities [26,27]. The observation of a significant association between increase in LST positivity and increasing age suggests a need to put in interventions to protect children in particular from exposure to *Leishmania* infection while additional studies are carried out to understand the reasons for the increased prevalence observed among the older segments of the study population [26,27].

Although *Leishmania* infection was observed in all three study communities, being a resident of Keri was significantly associated with increase in the likelihood of being exposed to the infection, while staying in Sibi Hill top has a less likelihood of being exposed to the infection, compared with being a resident of Ashiabre. This calls for more studies to better understand the characteristics of the study communities particularly with regards to determining how long members of the study communities may have been living with the health challenge of CL. This is important because of findings from other studies which observed higher prevalence of *Leishmania* infection in communities considered to be older/endemic foci for CL compared with those considered to be emerging foci [24,26].

Furthermore, observation of a significant association between being male and increased prevalence of *Leishmania* infection in the study communities calls for more studies to understand the daily activities of males in the study communities to develop appropriate measures to protect them from *Leishmania* infections. Also, the observation of a lack of statistical association between being LST positive and Never using ITN or use of ITN sometimes, when compared with those who used ITN often, suggests outdoor infection.

The factors associated with LST positivity in this study should therefore be taken into consideration in the development of future measure aimed at reducing exposure to Leishmania infection in the study area.

## Conclusions

This study has demonstrated exposure to *Leishmania* infection in three communities of the Oti region of Ghana using the LST. Being male, living in Keri and being five years or older were associated with an increase in the odds of exposure to *Leishmania* infection using LST. Future efforts aimed at reducing exposure to *Leishmania* infection in the study area should take the associated factors into consideration.

### Limitations of the study

The leishmanin skin test only indicates exposure to *Leishmania* and does not differentiate between recent and past exposures. A follow up study involving either culture of the parasite from a wound or molecular detection with sequencing will help to better characterize the infecting strains of *Leishmania* parasite. The use of questionnaire for this study may introduce some bias such as recall bias on the part of respondents. Furthermore, inclusion of a household in the study depended on the consent of the household head. This may have led to the exclusion of a few households, given that 587 households were included out of 600 households invited.

## Supporting information

S1 STROBE checklist Checklist according to The Strengthening the Reporting of Observational studies in Epidemiology *[STROBE]* guidelines. (DOCX)

S1 Individual Case Report form Study questionnaire. (DOCX)
