# Supplementary material for: Prevalence of Leishmania infection in three communities of Oti Region, Ghana
Source: PLoS Negl Trop Dis. 2021 May 27;15(5):e0009413. doi: 10.1371/journal.pntd.0009413 (PMC8158879; doi:10.1371/journal.pntd.0009413)
Supplement: S1 STROBE checklist — (DOCX) [file pntd.0009413.s001.docx]

STROBE Statement— Prevalence of Leishmania infection in three communities of Oti Region, Ghana

|  | **Item No** | **Recommendation** | **Page No.** | **Relevant text from manuscript** |
| --- | --- | --- | --- | --- |
| **Title and abstract** | 1 | (*a*) Indicate the study’s design with a commonly used term in the title or the abstract | 1 and 2 | Line 1; This cross-sectional study described prevalence of Leishmania infection in three communities in Oti Region, Ghana |
|  |  | (*b*) Provide in the abstract an informative and balanced summary of what was done and what was  found | 3 | Lines (38-46); Abstract |
| **Introduction** | | |  |  |
| Background/rationale | 2 | Explain the scientific background and rationale for the investigation being reported | 3 | Introduction (Lines 71-99) |
| Objectives | 3 | State specific objectives, including any prespecified hypotheses | 5 | Introduction Lines (96-99); This study was therefore conducted in three communities of the Oti Region (which until December, 2018 was part of the Volta Region) to investigate the prevalence of *Leishmania* infection following reports of cases of skin ulcers suspected to be CL. |
| **Methods** | | |  |  |
| Study design | 4 | Present key elements of study design early in the paper | 5 | Methods, Lines (105-109); Using a cross-sectional study design, this study was conducted in three communities of the Oti region of Ghana from October to December, 2018. |
| Setting | 5 | Describe the setting, locations, and relevant dates, including periods of recruitment, exposure, follow-up, and data collection | 6 | Methods, Lines (112 - 122); This study was conducted in the following three communities: Ashiabre, Keri, and Sibi Hilltop. Ashiabre is in the Tutukpene sub-district of the Nkwanta South municipality while Keri is in the Keri sub-district of the municipality |
| Participants | 6 | (*a*) *Cohort study*—Give the eligibility criteria, and the sources and methods of selection of participants. Describe methods of follow-up  *Case-control study*—Give the eligibility criteria, and the sources and methods of case | 5. |  |

|  |  | ascertainment and control selection. Give the rationale for the choice of cases and controls  *Cross-sectional study*—Give the eligibility criteria, and the sources and methods of selection of participants |  | Methods, Line 110 to 113; Eligible study participants were residents in the study community for > 12 months, aged between 2 to 65 years (inclusive). |
| --- | --- | --- | --- | --- |
|  |  | (*b*) *Cohort study*—For matched studies, give matching criteria and number of exposed and unexposed  *Case-control study*—For matched studies, give matching criteria and the number of controls per  case | N/A | This was a cross-sectional study |
| Variables | 7 | Clearly define all outcomes, exposures, predictors, potential confounders, and effect modifiers. Give diagnostic criteria, if applicable | 8 | Methods, Lines 166 - 176 |
| Data sources/ measurement | 8* | For each variable of interest, give sources of data and details of methods of assessment (measurement). Describe comparability of assessment methods if there is more than one group | 8. | Methods, Line 175 to 176; These variables were obtained from the administration of a structured questionnaire. |
| Bias | 9 | Describe any efforts to address potential sources of bias | 9 | Lines (177 to179); Odds ratios for all variables included in the multiple logistic regression analysis were adjusted for all covariates included in the model as well as for clustering at the household level using the vce (cluster clustvar) command in Stata statistical software version 14.  . |

|  |  |  |  |  |
| --- | --- | --- | --- | --- |
| Study size | 10 | Explain how the study size was arrived at | 6. | Methods. Line 131 to 134;  Assuming a 0.05 seropositivity of delayed-type hypersensitivity to *Leishmania* antigen in the study area, acceptable difference of 0.0175, an alpha error of 0.05, and a design effect of 1.5, a minimum sample of 834 persons was required for LST screening. |
| Quantitative variables | 11 | Explain how quantitative variables were handled in the analyses. If applicable, describe which groupings were chosen and why | 8 | Methods, Line 164 - 176;  Data were managed using Microsoft Access software version 2013 and analyzed using STATA software version 14. All statistical tested were performed at a 95% confidence level. |
| Statistical methods | 12 | (*a*) Describe all statistical methods, including those used to control for confounding | 8 | Methods, Line 166 to 169; |
|  |  | (*b*) Describe any methods used to examine subgroups and interactions | N/A | There were no subgroups |
|  |  | (*c*) Explain how missing data were addressed | N/A | There was no missing data |
|  |  | (*d*) *Cohort study*—If applicable, explain how loss to follow-up was addressed  *Case-control study*—If applicable, explain how matching of cases and controls was addressed *Cross-sectional study*—If applicable, describe analytical methods taking account of sampling strategy | 7 | Methods: **Selection of households for study inclusion,** Lines 136-143); Using a sorted list of households in each study community, 200 households (with an average of 5-7 persons per household) were selected for study inclusion using a systematic sampling approach. |
|  |  | (*e*) Describe any sensitivity analyses | N/A |  |
| Results |  |  |  |  |
| Participants | 13* | (a) Report numbers of individuals at each stage of study—eg numbers potentially eligible,  examined for eligibility, confirmed eligible, included in the study, completing follow-up, and analysed | N/A | This was a cross-sectional study with only one stage |
|  |  | (b) Give reasons for non-participation at each stage | N/A |  |
|  |  | (c) Consider use of a flow diagram | N/A |  |
| Descriptive data | 14* | (a) Give characteristics of study participants (eg demographic, clinical, social) and information on exposures and potential confounders | 9 | Results, Line 183 - 187;  A total of 3071 persons comprising 1091, 848, and 1132 participants from Ashiabre, Keri, and Sibi Hilltop respectively were screened using the LST procedure |

|  |  |  |  |  |
| --- | --- | --- | --- | --- |
|  |  | (b) Indicate number of participants with missing data for each variable of interest | N/A | There was no missing data |
|  |  | (c) *Cohort study*—Summarise follow-up time (eg, average and total amount) | N/A | This was a cross-sectional study |
| Outcome data | 15* | *Cohort study*—Report numbers of outcome events or summary measures over time | N/A | This was a cross-sectional study |
|  |  | *Case-control study—*Report numbers in each exposure category, or summary measures of exposure | N/A | This was a cross-sectional study |
|  |  | *Cross-sectional study—*Report numbers of outcome events or summary measures | 10-15. | Results, Line 191 to 253; |
| Main results | 16 | (*a*) Give unadjusted estimates and, if applicable, confounder-adjusted estimates and their precision (eg, 95% confidence interval). Make clear which confounders were adjusted for and why they were included | 14-15. | Results, Line 236 to 253. |
|  |  | (*b*) Report category boundaries when continuous variables were categorized | N/A | There were no continuous variables. |
|  |  | (*c*) If relevant, consider translating estimates of relative risk into absolute risk for a meaningful  time period | N/A | There were no estimates of relative risk |
| Other analyses | 17 | Report other analyses done—eg analyses of subgroups and interactions, and sensitivity analyses | N/A | There were no subgroups |
| **Discussion** |  |  |  |  |
| Key results | 18 | Summarise key results with reference to study objectives | 16-17. | Discussion, Line 257 to 287 |
| Limitations | 19 | Discuss limitations of the study, taking into account sources of potential bias or imprecision.  Discuss both direction and magnitude of any potential bias | 17. | Limitation, Line 296 to 298 |
| Interpretation | 20 | Give a cautious overall interpretation of results considering objectives, limitations, multiplicity of analyses, results from similar studies, and other relevant evidence | 17. | Conclusion, Line 290 to 293; This study has demonstrated exposure to Leishmania infection in three communities of the Oti region of Ghana using the LST. |

| Generalisability | 21 | Discuss the generalisability (external validity) of the study results | 17 | Discussion, Line 286-287; The factors associated with LST positivity in this study should therefore be taken into consideration in the development of CL control strategies in the study area |
| --- | --- | --- | --- | --- |
| **Other information** |  |  |  |  |
| Funding | 22 | Give the source of funding and the role of the funders for the present study and, if applicable, for the original study on which the present article is based | 18 | Lines 321-324; WHO/TDR post graduate training scheme fellowship in implementation science by the Special Programme for Research and Training in Tropical Diseases (WHO/TDR) at the School of Public Health, University of Ghana. The authors declare that the funder had no role in the study design, data collection, data analysis, data interpretation, and in writing the manuscript. |
