# Supplementary material for: Prevalence of Leishmania infection in three communities of Oti Region, Ghana
Source: PLoS Negl Trop Dis. 2021 May 27;15(5):e0009413. doi: 10.1371/journal.pntd.0009413 (PMC8158879; doi:10.1371/journal.pntd.0009413)
Supplement: S1 Individual Case Report form — (DOCX) [file pntd.0009413.s002.docx]

**QUESTIONNAIRE**

**Study title:** Leishmania infection and use of insecticide treated bed net (ITN) in selected communities of Volta region, Ghana **–**An implementation research

| individual case report | |
| --- | --- |
|  | |
| household information panel HH | |
| HH1A. Cluster number: ___ ___ ___  HH1B. Community name: ______________________ | HH2. Household number: __ __ __ \| __ __ __ |
| HH3. Interviewer name and number: | HH4. Supervisor name and number: |
| Name _________________________ ___ ___ | Name__________________________ ___ ___ |
| HH5. Day / Month / Year of interview: __ __ / __ __ / __ __ __ __ Study ID:  _______  HH Line#__ __ | |
| HH6. Area: Urban 1 Rural 2 | HH7. District: Nkwanta South 1  Nkwanta North 2  Ho 3  Other Specify 4 |

DOB (dd/mm/yyyy): ___ / ___ / _____ Age:____ Gender:  1. Male  2. Female

Occupation:  1. Farmer  4. Trader  7. Military

2. Logger  5. Student  8. Hunter

3. Teacher  6. Civil Servant  88. Other (specify): __________

| 1. Have you ever had any skin lesions?  0. No **GO TO 9**  1. Yes When? (mm/yyyy) ___/___  2. Did any of your previous skin lesions result in scars?  0. No  1. Yes  3. Do you think any of your skin lesions was due to cutaneous leishmaniasis?  0. No  1. Yes  99. Don’t know  4. Do you currently have any skin lesions?  0. No **GO TO 6**  1. Yes  5. When did this current skin lesion start? (mm/yyyy) ___/___  6. How many scars do you have currently?  ___ scars.  3. None  7. Have you ever treated any of your skin lesion(s)?  0. No  1. Yes  **8a. Where did you go for treatment? (Multiple answers)**  0. Nowhere (self-treatment)  1. Hospital  2. Traditional healer (self treatment)  3. Pharmacy  4. Other (Specify)…………………………. |
| --- |
| 8b. What did you use for treatment? **READ EACH RESPONSE AND MARK ALL THAT APPLY:**  1. Herbs  2. Hot stone  3. Dermacort Hydrocortisone cream (Observed)  7. No idea  7. Other (Specify)…………………………………  8c. How many days did the treatment last? _______ days  8d. Did you complete the treatment?  0. No Specify why not? ____________________  1. Yes  8e. What was the outcome?  1. Cured  2. Treatment failure  3. Relapsing |
| **PHYSICAL EXAMINATION**  9. Height: ______ cm 10. Weight: ______kg  11. Temperature: ____ ^o^C  1. Orally  2. Axillary  **12. Total number of suspected active lesions:** ___ lesion(s)  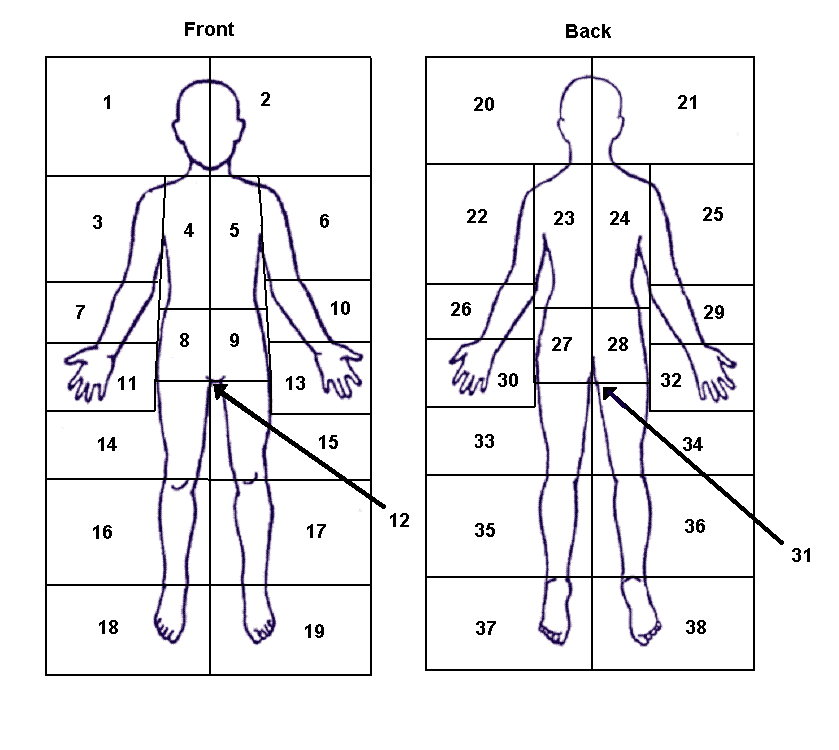**13. Complete the table with all the information from each lesion. Use the figure to localize the lesions on the patient’s body.**   \| **#** \| **Begin. Date (mm/yy)** \| **Type** \| **Localization (code)** \| **Size (mm)** \| **Sample taken (Y/N)** \| \| --- \| --- \| --- \| --- \| --- \| --- \| \| A \|  \|  \|  \| / \|  \| \| B \|  \|  \|  \| / \|  \| \| C \|  \|  \|  \| / \|  \| \| D \|  \|  \|  \| / \|  \| \| E \|  \|  \|  \| / \|  \|  \| **Type** \| **Sample taken** \| \| --- \| --- \| \| 1. Ulcer \| 1. Tissue aspirate \| \| 2. Nodule \| 2. Lancet sampling \| \| 3. Papule \| 3. Biopsy sampling \| \| 4. Plaque \| 4. Filter paper \| \| 5. Scar \|  \| |
| **III. EPIDEMIOLOGY**  14. Places of residence in the last 5 years (stayed more than 1 month)   \| **#** \| **From**  **(M/Y)** \| **To (M/Y)** \| **Region** \| **District** \| **Village** \| **Occup.** \| \| --- \| --- \| --- \| --- \| --- \| --- \| --- \| \| A \|  \|  \|  \|  \|  \|  \| \| B \|  \|  \|  \|  \|  \|  \| \| C \|  \|  \|  \|  \|  \|  \| \| D \|  \|  \|  \|  \|  \|  \| \| E \|  \|  \|  \|  \|  \|  \| \| F \|  \|  \|  \|  \|  \|  \| \| G \|  \|  \|  \|  \|  \|  \|   **Occupation:**   \| 1. Farmer \| 2. Logger \| 3. Teacher \| \| --- \| --- \| --- \| \| 4. Trader \| 5. Student \| 6. Civil Servant \| \| 7. Military \| 8. Hunter \|  \| \| 88. Other (specify):__________ \|  \|  \| \|  \|  \|  \| |
| **Please, answer the following questions about your practices and behaviors during your daily routine: MARK ONLY ONE ANSWER PER QUESTION:**  1 2 3 4 5  \|_________\|________\|________\|__________\|  Never Rarely Sometimes Often Always   \|  \| **Rating** \| **Does not apply** \| \| --- \| --- \| --- \| \| 15. Do you use mosquito repellent? \|  \|  \| \| 16. Do you use long sleeves \|  \|  \| \| 17. Have contact with dogs \|  \|  \| \| 18. Have contact with goats? \|  \|  \| \| 19. Do you have contact with other domestic animals \|  \|  \| \| 20. Have you been at open field at dawn or sunset \|  \|  \| \| 21. Do you sleep in bed nets \|  \|  \| \| 22. Do you sleep in rooms or places with open windows without screen? \|  \|  \| \| 23. Do you sleep in areas near to forest or farm fields? \|  \|  \| \| 24. Did you spray your bedroom with insecticide in the last six months? \|  \|  \| \| 25. Have you had mosquito bites \|  \|  \| \| 26. Have you had bites from other insects? \|  \|  \| |
| 27. Specify any insects which has bitten you (other than mosquitoes) in the last six months?  ……………………………………………………………….  ……………………………………………………………….  **Does not apply** ……………………….3 |
|  |
| **IV. LABORATORY (Q29-33 ONLY FOR THOSE WITH SUSPECT CL LESION)**   \|  \| **Result** \| \| --- \| --- \| \| 28. Malaria RDT \|  \| \| 29. Direct observation \|  \| \| 30. Culture \|  \| \| 31. LST \|  \| \| 32. Real time PCR \|  \|   **Result:**  0. Negative  1. Positive  99. Not performed |
| 33 *Leishmania* species causing the current episode:  1. *L. major*  2. *L. tropica*  3. *L. ethiopica*  98. Unknown |
